# Jejunal hemolymphangioma: A case report

**DOI:** 10.1097/MD.0000000000018863

**Published:** 2020-01-24

**Authors:** Yajie Teng, Jie Wang, Qinhua Xi

**Affiliations:** Department of Gastroenterology, The First Affiliated Hospital Of Soochow University, Soochow, Jiangsu, China.

**Keywords:** benign tumor, double-balloon enteroscopy, hemolymphangioma, small intestine

## Abstract

**Rationale:**

Hemolymphangioma is a benign tumor comprised of the newly-formed lymph spaces and blood vessels, which can usually be found in the head and neck of the affected children. There are few reports regarding cases with hemolymphangioma in small intestine, spleen, esophagus, and other organs.

**Patient concerns:**

Herein, a 55-year-old woman was presented in this study, she had complained of discomfort in the right upper abdomen for 2 months, and was discovered with a space-occupying lesion in proximal jejunum on computed tomography (CT). Eventually, the lesions were confirmed through double-balloon enteroscopy (DBE) to be located in the jejunum 60 cm away from the Treitz ligament.

**Diagnose:**

Subsequently, the small intestine was partially resected, and postoperative pathology had confirmed the diagnosis of small intestinal hemolymphangioma.

**Interventions:**

Excisional surgery of the lesion was planned. On surgery, the lesions were discovered to be about 3^∗^3 cm to 2^∗^2 cm when engorged the superficial vessels. No enlarged lymph nodes were seen at the root of the mesentery, and no obvious lesion was observed in the remaining small intestine.

**Outcomes:**

Follow-up for 6 months showed no recurrence.

**Lessons:**

Hemolymphangioma lacks typical clinical symptoms, and the correct preoperative diagnosis of hemolymphangioma remains challenging. Due to the increasing use of endoscopic diagnostic techniques, it is expected that hemolymphangioma in gastrointestinal tract may be detected and endoluminal located before surgery more feasibly. This case report aimed to highlight the contributions of CT and DBE to an accurate preoperative diagnosis and surgical strategy planning.

## 1 Introduction

Hemolymphangioma is a rather rare benign tumor in the vascular system, which is characterized by the cystically dilated lymphatics.^[1]^ Histologically, hemolymphangioma consists of both the blood vessels and the lymphatic channels. Hemolymphangioma is usually discovered in the head, axilla, and neck, but rarely in the gastrointestinal tract, especially in the small intestine. Only a limited number of hemolymphangioma cases have been reported in literature thus far due to its low morbidity, and the correct preoperative diagnosis of hemolymphangioma remains challenging. This paper had reported a case with jejunal hemolymphangioma discovered by computer tomography (CT), localized by double-balloon enteroscopy (DBE), and finally managed with surgical resection.

## 2 Case report

**Figure 1:**
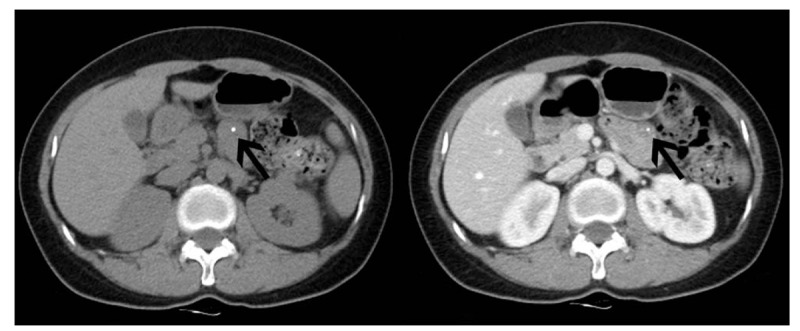
CT demonstrated a space-occupying lesion in proximal jejunum with calcium deposition, which had exhibited enhancement after contrast injection. CT = computed tomography.

**Figure 2:**
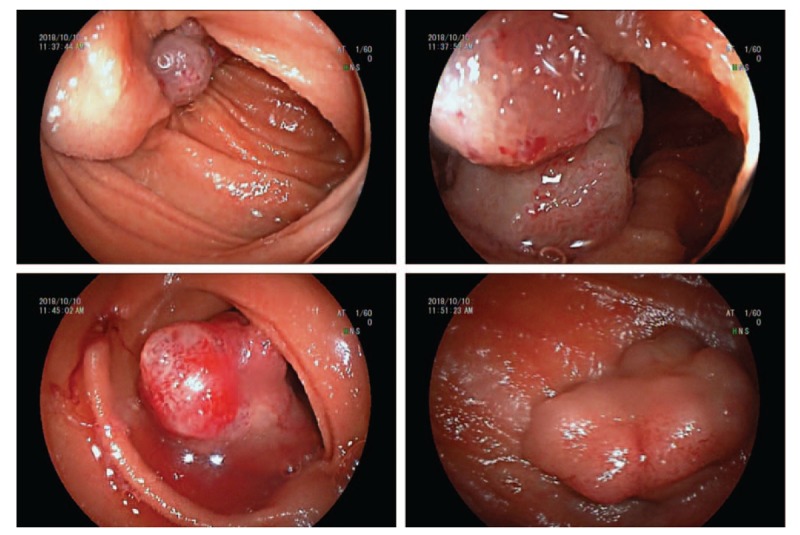
The DBE showed 2 congested and ulcerated polypoid masses in the proximal jejunum 60 cm away from the Treitz ligament. DBE = double-balloon enteroscopy.

A 55-year-old women was admitted into the First Affiliated Hospital of Soochow University as a result of complaint of discomfort in the right upper abdomen for 2 months. The patient also had a history of abdominal fullness and weakness, but she denied the symptoms of nausea, vomiting and fever, and had no medical history of abdominal trauma or surgery, no family history of malignant tumor, and no weight loss. Physical examination revealed mild tenderness on the right upper abdominal quadrant, with no rebound tenderness, and no abdominal mass was detected. No abnormality was detected in laboratory examinations, including blood routine examination, biochemical examination, and tumor makers. CT in other hospital demonstrated a space-occupying lesion in proximal jejunum with calcium deposition, which had exhibited enhancement after contrast injection (Fig. 1). Radiologically, it was diagnosed as jejunal space-occupying lesion and differential diagnosis of polyps and malignancy lesions. Therefore, the patient was referred to our department for small intestinal endoscopy. The DBE showed 2 congested and ulcerated polypoid masses in the proximal jejunum 60 cm away from the Treitz ligament (Fig. 2); as a result, enteroscopy was performed to sample for pathological analysis. Results of biopsy revealed chronic inflammation with low-grade intraepithelial neoplasia in the focal gland.

**Figure 3:**
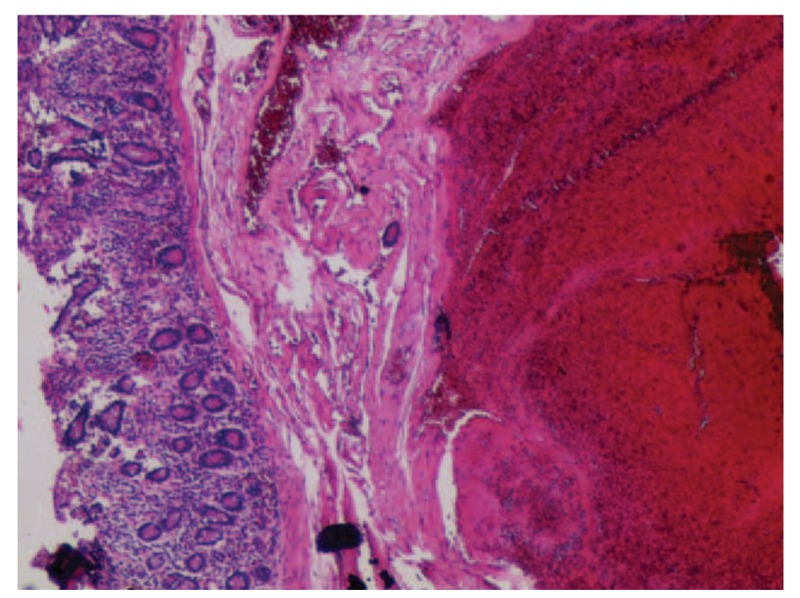
Hematoxylin and eosin staining of the case supported a diagnosis of hemolymphangioma. Magnification, ×40.

The patient was then transferred to the General Surgery Unit of our hospital in October 2018. She had undergone laparotomy, and the 2 lesions were found to be located in the jejunum 60 cm away from the Treitz ligament intraoperatively, at an interval of 3 cm. Afterward, the lesions were discovered to be about 3 ^∗^3 cm to 2^∗^2 cm when engorged the superficial vessels. No enlarged lymph nodes were seen at the root of the mesentery, and no obvious lesion was observed in the remaining small intestine. Subsequently, the small intestine was partially resected, and postoperative pathology had confirmed the diagnosis of small intestinal hemolymphangioma (Fig. 3). The postoperative course of the patient was uneventful and the patient was discharged 7 days after the surgery. No complicated or recurrence was noted during 6 months of follow-up. This study was approved by the Medical Ethics Committee of the First Affiliated Hospital of Soochow University. Written consent for this case report was obtained from the patient.

## 3 Discussion

Hemolymphangioma is a benign hamartoma of blood and lymphatic vessels, which originates from the mesenchymal tissue.^[2]^ In general, hemolymphangiomas refer to the large masses of cystic cavities with varying sizes and thin walls. They have multiple thin septa; besides, they are hemorrhage-like and are rarely of clear lymphatic nature.

Hemolymphangioma can be divided into primary and secondary lymphatic vascular tumors. Of them, the former is considered as a congenital malformation of the lymphatic vascular system, which is possibly formed due to the obstruction of the veno-lymphatic communication between dysembryoplastic vascular tissue and the systemic circulation. By contrast, the latter is induced by poor lymph drainage and lymphatic damage as a result of surgery or trauma.^[2,3]^

The clinical manifestations of hemolymphangiomas can vary in size and location. The symptoms vary depending on either the pressure on the adjacent structures is caused by the enlarging mass, or by the complications such as hemorrhage, infection, perforation, torsion, and rupture.^[4]^ Typically, clinical diagnosis of hemolymphangioma is rare, which can be ascribed to its low morbidity and the lack of clinical expression. In our case, the patient had the main complaints of abdominal discomfort with fullness and weakness, which might be induced by the space-occupying tumor.

Hemolymphangioma in gastrointestinal tract is clinically diagnosed based on CT, magnetic resonance imaging (MRI) and Endoscope. Specifically, CT and MRI are the useful tools to define the tumor extent and invasion, and to plan the surgical strategy.^[5]^ CT scan can be very helpful in radiological diagnosis. Hemolymphangioma shows a combined architecture of dilated vein, dilated lymphatics, and normal stromal tissue and normal vasculature in between. Malformed and dilated venous vessels are usually presented with thrombosis that can form dystrophic necrosis and calcium deposition.^[6]^ Notably, the different sizes of blood vessels in hemolymphangioma may lead to different enhanced characteristics upon imaging. Significant and persistent enhancement can be observed in tumors rich in blood vessels; meanwhile, a septum may also display marked enhancement.^[5,7]^ Typically, MRI can assist in determining the association of hemolymphangioma with the surrounding tissues, as well as the extent of invasion.^[7]^ Capsule endoscopy allows for displaying the pathological changes in the small intestine, which is associated with the advantages of no pain and safety, as well as the risk of capsule retention in the presence of oversized mass. However, it is difficult to diagnose an endoluminal localization. In comparison, DBE allows for the simultaneous biopsy and endoscopic treatment^[8]^; more importantly, the pathological changes are much clearer under DBE. Nonetheless, a definitive diagnosis should always be based on histological evidence, while biopsy should not be performed because of its high risk of massive bleeding.^[9]^ In our case, endoscopic biopsies were performed, and bleeding could be observed afterward, so local spraying with norepinephrine was prescribed, which could successfully stop the bleeding. Notably, the rich vascular tissues in hemolymphangioma may account for the cause of hemorrhage after biopsy.

Generally, complete excision can provide the best results with a lower recurrence rate; however, careful follow-up is necessary.^[10]^ The recurrence rate varies depending on the complexity, anatomical location, and adequacy of the excision. According to literature reports, 10% to 27% of the completely excised lesions would recur, whereas 50% to 100% of the partly resected tumors may recur. Compared with surgical treatments, nonsurgical treatments, including cryotherapy, laser therapy, radiotherapy, and local injection of sclerotic agents, are not superior.^[11]^

In conclusion, the current case report has presented a hemolymphangioma lesion in the small intestinal of a female patient. Hemolymphangioma lacks typical clinical symptoms, and specific radiological findings such as CT and MRI are helpful in confirming the diagnosis and appropriating treatment selection. Endoscopic diagnostic techniques for small intestinal hemolyphangioma also contribute a lot to an accurate preoperative diagnosis and surgical strategy planning. Although the hemolymphangioma is a very rare benign tumor, especially involving the small intestine. The possibility of hemolymphangioma should be taken into account when patients have repeated and unexplained gastrointestinal symptoms. Radiology and endoscopy are very significant for its diagnosis and treatment.

## Author contributions

**Resources:** Qinhua Xi.

**Writing – original draft:** Yajie Teng.

**Writing – review & editing:** Jie Wang, Qinhua Xi.
